# Ovarian Steroid Cell Tumor (Not Otherwise Specified): A Case Report of Ovarian Hyperandrogenism

**DOI:** 10.1155/2020/6970823

**Published:** 2020-04-08

**Authors:** Hadjkacem Faten, Ghorbel Dorra, Charfi Slim, Safi Wajdi, Charfi Nadia, Chaabene Kais, Boudawara Tahia, Abid Mohamed

**Affiliations:** ^1^Endocrinology Department, Hedi Chaker Hospital, 3029 Sfax, Tunisia; ^2^Department of Anatomical Pathology and Cytology, Habib Bourguiba Hospital, Sfax, Tunisia; ^3^Department of Gynecology, Hedi Chaker Hospital, 3029 Sfax, Tunisia

## Abstract

Steroid cell tumors (SCTs) (not otherwise specified (NOS)) are rare sex cord-stromal tumors of the ovary. These are associated with hormonal disturbances resulting in menstrual bleeding patterns and androgenic effects. We report the case of a 36-year-old female presented with hirsutism, signs of virilization, and elevated androgen levels. Transvaginal ultrasound showed a solid-appearing right ovarian mass. She underwent fertility-sparing surgery with a laparoscopic left oophorectomy. Histological examination showed a benign steroid cell tumor, NOS. These tumors often small can then present a problem of positive diagnosis responsible for a delay in the diagnosis.

## 1. Introduction

Steroid cell tumors (SCTs) (not otherwise specified (NOS)) are tumors of the ovary that cannot be characterized as stromal luteomas or Leydig cell tumors. SCT accounts for 0.1% of ovarian neoplasm. About 80% of SCTs fall into the NOS category [1]. They are androgenic tumors that may be associated with paraneoplastic manifestations such as hypercalcemia, erythrocytosis, or ascites. In 20% of the cases, the tumor cells will have already spread to contiguous organs at the time of surgery. The tumors with microscopic cellular pleomorphisms, high mitotic activity, or necrosis should be considered malignant [2].

## 2. Case Report

We report the case of a 36-year-old woman who presented with virilizing symptoms and secondary amenorrhea. The family history was negative for any type of cancer or sterility. She did not take any medication. She suffered from primary sterility for 11 years. The patient's history revealed that menarche had occurred at the age of 13 years and that regular menses lasting 5 to 6 days had occurred every thirty days since then. The patient reported that the excess of hair had started at the age of 25 years with progressive aggravation. The patient has had amenorrhea for several months after a long period of irregular menstrual cycles.

Physical examination revealed a normotensive female with a body mass index (BMI) of 21 kg/m^2^, with obvious facial hair that required weekly chin and daily lip shaving. Excessive hair was present on her chest, lower abdomen, and thighs giving a score of 22 according to the modified Ferriman and Gallwey scoring system. In the pelvic examination, an enlarged clitoris was notable. She also had temporal and parietal hair recession, male pattern baldness, alopecia, and deepening voice. The breasts looked normal developed (Tanner 5). Laboratory analysis revealed a high level of total serum testosterone: 1.71 ng/ml (normal 0.3–0.75 ng/ml) and delta-4-androstenedione: 4.1 ng/ml (normal 0.4–3.5 ng/ml). A 17-hydroxyprogesterone level was normal after adrenal stimulation (8.4 < 10 ng/ml). She had normal values of dehydroepiandrosterone sulfate, prolactin, cortisol, thyroid-stimulating hormone (TSH), and human chorionic gonadotropin (Table 1). Tumor markers including serum CA 125, carcinoembryonic antigen (CEA), CA 19–9, CA 15–3, and a-fetoprotein (AFP) were normal.

No adnexal masses were found using a transvaginal ultrasound (US) scan of the pelvis. Therefore, the diagnosis of polycystic ovary syndrome (PCOS) was established, based on the modified criteria of Rotterdam 2003 when two criteria were found: anovulation with clinical and biochemical evidence of androgen excess after the exclusion of other related disorders.

Laparoscopic examination of the ovaries was performed, including an ovarian biopsy and drilling. The pathological examination of ovarian biopsies did not suggest any specific pathology. The postoperative course was marked by an increase in serum testosterone to 10 ng/ml. This level had prompted the search for tumors secreting androgens. The controlled pelvic US showed that the left ovary contained multiple small follicular formations while the right ovary had an increased size caused by a hyperechoic homogeneous ovarian cyst measuring 42 × 34 mm. The magnetic resonance imaging (MRI) revealed the absence of an adrenal mass and the presence of multiple cystic formations of the two ovaries, one of them being hemorrhagic with a size of 23 mm. Therefore, an ovarian tumor was suspected. The patient underwent a second exploratory laparotomy. A cystic mass in the right ovary was removed with biopsy of the left ovary and cytological examination of peritoneal fluid.

**Figure 1:**
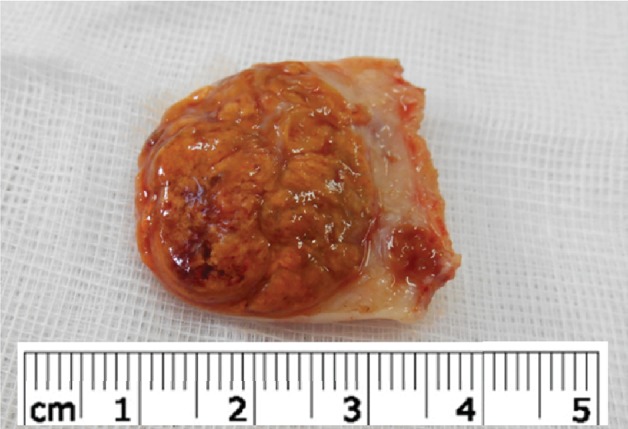
Lobulated mass of the ovary with yellow-orange cut surface.

Pathological examination of the surgical specimen revealed an enlarged right ovary, with a well-circumscribed mass without vegetation on the ovary surface measuring 45 × 45 × 35 mm. The cut section of the specimen showed a lobulated and yellow-orange color (Figure 1).

**Figure 2:**
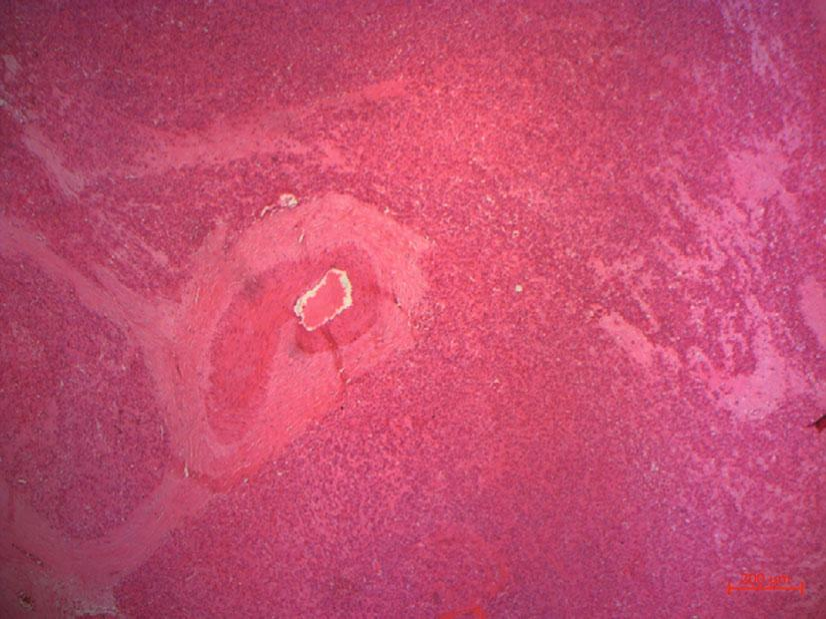
A diffuse tumor cell proliferation with fibrous septa (hematoxylin eosin 40x).

**Figure 3:**
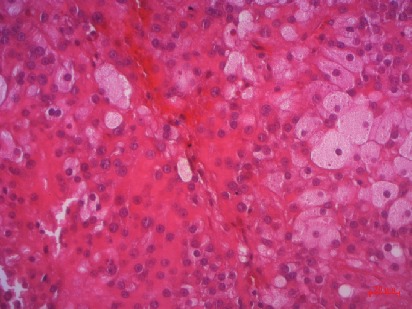
Tumor cells were polygonal with granular eosinophilic cytoplasm. They were intermingled with some large pale and vacuolated cells (hematoxylin eosin 40x).

Microscopically, tumor cells were arranged in a diffuse pattern separated by fibrosis septa. Tumor cells were large, polygonal with abundant granular eosinophilic cytoplasm. Nuclei were round with prominent central nucleolus. There is no significant cellular pleomorphism. These cells were intermingled with large cells with vacuolated and pale cytoplasm (Figures 2 and 3).

**Figure 4:**
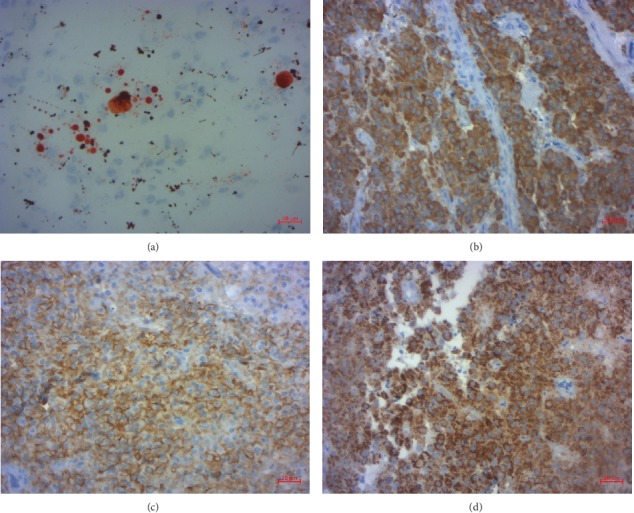
(a) Oil-red-O stain demonstrates numerous lipid droplets (40x); (b) positive staining of tumor cells for inhibin (400x); (c) positive staining of tumor cells for CD56 (400x); (d) positive staining of tumor cells for Melan A (400x).

Crystals of Reinke were not seen. The staining of Oil-red-O has revealed numerous intracellular lipid droplets (Figure 4(a)). No mitotic figures were identified. No hemorrhagic or necrotic contents were noted. Margins of the resected specimen were positive for tumor cells. Immunohistochemical study showed strong and diffuse positivity of tumor cells for inhibin, CD56, calretinin, and Melan A (Figures 4(b)–4(d)).

Tumor cells were negative for keratin, synaptophysin, chromogranin, and hepatocyte cell. Immunoreactivity for Ki-67 (proliferative index) was noted in less than 1% of all tumor cells. A benign ovarian steroid tumor, NOS, was confirmed. Biopsy of the left ovary showed numerous follicular cysts with no tumor infiltration. Cytological smears of the peritoneal fluid were negative for tumor cells. A complete surgical staging procedure was performed consisting of a right oophorectomy with biopsy of the left ovary, the paracolic gutters, and the pouch of Douglas. Anatomopathology examination concludes that there was no tumor residue in the right ovary. The right tube, the left ovarian biopsy, and the omentectomy piece were free of abnormalities. The final surgical-pathological stage of the SCT of the ovary was Ιa, without extension and/or metastases to the uterus and/or tubes. No adjuvant therapy was used.

The patient has been followed up for 1 year since the surgery, without evidence of recurrence or metastasis on clinical, biological, and radiological examinations. She reported that her facial and truncal hair growth has decreased markedly and that she no longer suffered from losing hair from the temporal areas of her scalp. She resumed her menstrual cycles within 2 months of the operation. The serum testosterone and the androstenedione level returned as normal (Table 1).

## 3. Discussion

The term (SCT; NOS) was first used by Scully in 1979 and describes a particularly rare type of ovarian sex cord-stromal tumors, which comprise <0.1% of all ovarian tumors. These tumors were previously classified as lipid or lipoid cell tumors. There are steroid cell tumors that cannot be categorized as either stromal luteomas or Leydig cell tumors. This group of tumors accounts for 60% of all ovarian steroid cell tumors [3–5].

These tumors generally possess little or no cytoplasmic lipids. Therefore, it is difficult to discriminate them from other oxygen or clear ovarian cell tumors. That is the reason why the association of the different clinical, histological, and immunohistochemical characteristics is necessary to assure finding the right diagnosis [6].

The tumors can occur at any age with a mean age of 43 years, which is younger than other steroid tumors. They rarely occur before puberty [4]. Among the patients affected with SCT-NOS, 56-77% have symptoms of androgenic changes, such as hirsutism and virilization including acne, clitoral enlargement, deep voice, and alopecia. 6-23% have estrogenic manifestations such as menorrhagia, postmenopausal bleeding, or even endometrial carcinoma [4]. Only 6-10% are clinically associated with Cushing's syndrome, and 25% of SCT-NOS are nonfunctioning [5].

Investigation of severe hyperandrogenism probably caused by an ovarian neoplastic source is a major diagnostic challenge in some circumstances. In the case of a young woman, serum 17-hydroxyprogesterone should be obtained to screen for late-onset congenital adrenal hyperplasia [7]. In virilizing patients, serum testosterone levels higher than 2 ng/ml, normal dehydroepiandrosterone-sulfate, and no evidence of 21-hydroxylase deficiency are strong evidence of the diagnosis of ovarian virilizing tumor or ovarian hyperthecosis [8]. Various other serum tumor markers (including AFP and CA-125) facilitate the differential diagnosis of ovarian adenocarcinoma [9].

Some of these tumors are so small that they may stay undiagnosed even after careful radiological scrutiny. Furthermore, no radiological characteristics of the tumors of steroid cells have been reported in the literature.US imaging provides little conclusive evidence to diagnose SCT of the ovary as it may be misinterpreted as follicular growth [8]. Transabdominal and vaginal US can be useful in the evaluation of the ovarian size and morphology of the ovary [7]. MRI is useful for identifying small solid ovarian tumors, although findings may be unspecific in the case of virilizing ovarian tumors. However, this imaging is still an effective method for preoperative staging of SCT of the ovary and for defining the differential diagnosis including polycystic ovary syndrome. The characteristic MRI appearance of polycystic ovaries consists of bilateral enlarged ovaries with multiple small subcapsular cysts and prominent central stroma [8]. The small tumor size may explain the initial diagnosis delay in our case. Therefore, finally, if no obvious radiological mass is found, percutaneous sampling of the adrenal and ovarian vessels can help to localize small steroid-producing tumors [7].

The majority of these tumors have benign behavior or like low-grade tumors. However, clinically malignant behavior occurs in 25-40% of the patients usually within the peritoneal cavity and rarely at distant sites [6, 10]. Most of these cases are unilateral tumors, only 6% of the cases are found to be bilateral [4].

Steroid cell tumors are typically solid and well-circumscribed, occasionally lobulated, with a mean diameter of 8.4 cm. They are solid and maybe yellow-orange, red, brown, or black. Hemorrhage is more common than in cases of Leydig cell tumor [1]. World Health Organization (WHO) Classification of Tumours of Female Reproductive Organs defined SCT as a tumor which is composed entirely of cells resembling steroid-secreting cells that lack Reinke crystals [1]. Steroid cell tumors often resemble cells of the adrenal cortex, although most of them are probably of ovarian stromal origin. However, adrenal and ovarian steroid-producing cells are both derived from a common primitive mesenchymal cell. It can also be argued that when these cells become neoplastic, previously repressed genes become functional, resulting in the subsequent activation of enzyme systems. Therefore, these cells start to function like adrenocortical tissue.

Microscopically, these tumors are composed of polygonal cells with abundant vacuolated or eosinophilic cytoplasm [11, 12]. In histopathology, the cells are most commonly arranged in a diffuse pattern but can grow in nests or cords. Stroma may range from scant to prominent with fibrous bands. The tumor cells are polygonal and have abundant cytoplasm that ranges from eosinophilic (lipid-poor) to pale and vacuolated (lipid-rich); the latter is much more common than in Leydig cell tumor. Steroid cell tumors, NOS, differ from Leydig cell tumors (hilar and nonhilar type) in the lack of crystals of Reinke in the cytoplasm. Variable amounts of intracytoplasmic lipochrome can be present. The nuclei are typically round with a prominent central nucleus; rarely, there is substantial nuclear atypia, usually accompanied by increased mitotic activity. In some small SCT, a small stromal hyperthecosis is particularly seen in the adjacent ovarian stroma [1].

Hayes and Scully have identified five pathological predictive characteristics of malignancy as follows: two or more mitotic figures/ten high-power fields (malignancy in 92%), necrosis (malignancy in 86%), a diameter of more than 7 cm (malignancy in 78%), hemorrhage (malignancy in 77%), and grade 2 or 3 of nuclear atypia (malignancy in 64%) [13].

By immunohistochemistry, these tumors are positive for sex cord-stromal markers, such as inhibin, calretinin, and steroidogenic factor-1. They are usually positive for Melan A and negative for FOXL2 [1]. Of the different markers that have been reported to stain these tumors, inhibin has proven to be the most helpful to date, because most steroid cell tumors express this marker [14]. In our case, the tumor showed strong and diffuse positivity of tumor cells for inhibin and Melan A.

The first-line treatment is surgery. In a young woman with stage ΙA disease, for whom fertility is an issue, only unilateral salpingo-oophorectomy needs to be performed. Although, in older women, for whom maintaining fertility is not necessary, total abdominal hysterectomy, bilateral salpingo-oophorectomy, and complete surgical staging should be carried out [8]. Nevertheless, adjuvant chemotherapy should be based on the histologic appearance of the tumor and on its surgical stage [7].

Malignant SCT-NOS should be managed with total abdominal hysterectomy, bilateral salpingo-oophorectomy, and sampling of pelvic and mesenteric lymph nodes and of the omentum followed by combination chemotherapy. Since a definitive chemotherapy regimen is not yet defined, bleomycin, etoposide, and cisplatin (BEP) are favored and frequently used. Chemotherapy may be applied using the BEP regimen (bleomycin (20 U/m^2^ every 3 weeks for 4 cycles), etoposide (75 mg/m^2^ on days 1-5, every 3 weeks for 4 cycles), and cisplatin (20 mg/m^2^ on days 1-5, every 3 weeks for 4 cycles)) or other medications [7]. To date, very few reports have investigated the efficacy of radiation or chemotherapy for SCTs [10].

Additionally, gonadotropin-releasing hormone agonists have been used as therapy for recurrent malignant disease exploiting a suppressive effect on ovarian steroidogenesis [4].

Finally, a careful follow-up evaluation should include a measurement of sex hormone levels, particularly for those patients who demonstrated elevated levels before removal of the primary tumor [15].

## 4. Conclusion

This case underscores that SCT, NOS, is among the rarest ovarian tumors and therefore difficult to diagnose. Careful history taking, physical examination and laboratory testing, in addition to imaging studies, and laparoscopic examination are helpful in making the diagnosis. Treatment should be individually based on tumor pathological and histological features, surgical staging, and the desire for future fertility [7, 16].

We recommend that any female patient who presents with high testosterone levels should be investigated systematically in order to determine whether the origin could be adrenal or ovarian. An awareness of this entity will extend the appreciation of NOS ovarian steroid cell tumors.

## Figures and Tables

**Table 1:** Reproductive endocrine hormone levels before and after surgery.

	FSH (mUI/ml)	LH (mUI/ml)	E2 (pg/ml)	PRL (ng/ml)	T (ng/ml)	DHEA-S (*μ*mol/l)	Delta4An (ng/ml)
Normal values of age	1.5-12.4	1.7-8.6	12.4-233	4-15	0.3-0.75	2.7-9.2	0.4-3.5
Before surgery	5.9	5.5	28	10.9	1.7	8.3	4.1
After ovarian drilling	5	8.83	47	14.8	10	UV	UV
1 month after surgery	5.5	5.6	30.2	19.19	0.13	5.6	1.9
1 year after surgery	6.7	7.3	48.6	16	0.16	UV	UV

T: testosterone; Delta4An: delta-4-androstenedione; DHEA-S: dehydroepiandrosterone sulfate; FSH: follicle-stimulating hormone; LH: luteinizing hormone; E2: oestradiol; PRL: prolactin; UV: uncontrolled value.
